# 

**Published:** 1897-04

**Authors:** 


					﻿CONTENTS.
ORIGINAL ARTICLES—
Congestion of the Uterus, Its .Causes and Treatment. By John
Thompson Graham, M. D., Wytheville, Va’.‘.................. 155
A Few Notes on the Morbid Anatomy and Pathology of Varix
and Allied Affections, Especially as They Occur in the Veins of
the Lower Extremities. By Thomas H. Manley, M. D., New
York........................................................ 160
Some Dottings on Hemorrhage. By J. McFadden Gaston, M. D.,
Atlanta............................ '......... •.............. 170
Multiple Bilateral Cystoma of the “Organ of Giraldes.” By Wil-
liam C. Dugan, M. D., Louisville, Ky....................... 171
SELECTIONS AND ABSTRACTS—
Condensed Facts Concerning Electricity for the General Practi-
tioner...............................................       173
The Diagnosis and Treatment of Lumbago...................... 175
Bullets in the Brain and the Roentgen Rays................'.	176
Uncommon Affections of the Eyelids.......................... 177
Unconsciousness from Constipation   ...................... 170
Emile Zola as a Degenerate.........................’........ 180
Microscopical Note...................................    181
Morphine—Ether Narcosis.	...................   182
Dangers of Morphine...................................   182
Hernia............................................       183
Renipuncture in Albiminuria...........................   184
Aotion, Application and Value of Oleum Bucalypti e Follis (Eu-
calyptol.........................................        185
Beef Essence............................................ 188
Treatment of Pott’s Curvature.......................     188
A Curious Case of Religious Monomania. >	................... 189
Contagious Impetigo. 11...........1..................... 189
The Comparative Value of Eucaine and Cocaine as Anesthetics.. 190
A New Suture Material.................................   191
Skiagraphs in the Diagnosis of Thoracic Disease......... 192
The Cistern—A Substitute for the Country Well........... 193
Treatment of Placenta Previa............................ 195
EDITORIAL...............................// .. .............. 196
SPECIAL NOTES.............................................   200
PRESCRIPTIO NS............................................   207
				

